# S229. CAN LONG-ACTING INJECTABLE PALIPERIDONE DOSING BE OPTIMIZED WITH PLASMA LEVEL MEASUREMENTS?

**DOI:** 10.1093/schbul/sby018.1016

**Published:** 2018-04-01

**Authors:** Robert Zipursky, Helen Huynh, Ofer Agid, Michael Kiang, Gary Remington

**Affiliations:** 1 McMaster University; 2 St. Joseph’s Healthcare Hamilton; 3 Centre for Addiction and Mental Health (CAMH), University of Toronto

## Abstract

**Background:**

Most people with schizophrenia respond robustly to antipsychotic medication but are at very high risk of relapse if these medications are stopped. Long-term maintenance treatment with antipsychotic medication can dramatically reduce the risk of relapse. With long-acting injectable antipsychotic medication (LAI), adherence is documented which may account for superior efficacy in relapse prevention reported in some studies. It is known that plasma antipsychotic levels vary greatly across individuals with standard doses of LAIs. Establishing the lowest effective plasma levels for relapse prevention may also help in minimizing side effects that may contribute to problems with adherence. This study was carried out to describe the plasma paliperidone levels associated with clinical stability in patients receiving the LAI, paliperidone palmitate. We predicted that higher paliperidone plasma levels would be associated with lower subjective well-being and greater levels of sexual dysfunction.

**Methods:**

Patients with clinical diagnoses of schizophrenia and schizoaffective disorder attending specialized schizophrenia outpatient clinics at St. Joseph’s Healthcare Hamilton were invited to participate if they were receiving maintenance treatment with paliperidone palmitate. The study involved two visits, 3 to 4 weeks apart, on days that subjects were scheduled to receive consecutive injections of paliperidone palmitate. Plasma paliperidone levels and prolactin levels were drawn prior to the injection at Visit 1 and a second paliperidone levels was drawn at Visit 2. At Visit 1, a series of rating scales were also completed including the Subjective Well-being under Neuroleptic scale – Short version (SWN), the Changes in Sexual Functioning Questionnaire (CSFQ) and the Drug Attitude Inventory (DAI).

**Results:**

Twenty-one subjects (11F/10M) provided informed consent for this study and had plasma paliperidone levels measured. Patients had been receiving LAI paliperidone for a mean of 18 months (SD = 11.4). Mean paliperidone levels at Visit 1 (n=21) and Visit 2 (n=18) were 34.9 ng/ml (SD = 20.0 ng/ml; range = 5.1–73.9 ng/ml) and 35.1 ng/ml (SD = 17.2 ng/ml; range = 9.0–67.5 ng/ml), respectively. Plasma paliperidone levels measured at Visit 2 were highly correlated with levels from Visit 1 (n=18; r = .89, p <.001). Plasma prolactin levels were correlated with levels of plasma paliperidone (n=21, r=0.56, p < .01). Lower scores on the CSFQ – Sexual Desire factor were associated with higher levels of paliperidone (n=19, r =-.61, p<.01) and prolactin (n=19, r=-.56, p <.01). Higher paliperidone levels were associated with more negative scores on the Drug Attitude Inventory (n=19, r=-0.49, p < .05). Plasma paliperidone levels were not associated with scores on the SWN (n=21, r=-.-02).

**Discussion:**

In patients receiving maintenance treatment with paliperidone palmitate, plasma paliperidone levels varied approximately 15-fold. Higher paliperidone levels were associated with more negative attitudes towards medication and more severe deficits in sexual desire but not with subjective well-being. Many stable patients had plasma level close to the 20ng/ml level which in PET studies leads to 65% dopamine D2 receptor occupancy, a level reported to be associated with antipsychotic response. Our findings raise the possibility that maintaining patients at levels just above the 20ng/ml level may be sufficient for relapse prevention but may spare the adverse effects such as sexual dysfunction associated with higher plasma levels. These results suggest that measuring plasma levels in patients receiving paliperidone as a LAI may be of value in identifying the minimum effective dose for prevention of relapse and side effects.

